# Prevalence, sensitivity and specificity of antibodies against carbamylated proteins in a monocentric cohort of patients with rheumatoid arthritis and other autoimmune rheumatic diseases

**DOI:** 10.1186/s13075-016-1173-0

**Published:** 2016-11-25

**Authors:** Arbi Pecani, Cristiano Alessandri, Francesca Romana Spinelli, Roberta Priori, Valeria Riccieri, Manuela Di Franco, Fulvia Ceccarelli, Tania Colasanti, Monica Pendolino, Riccardo Mancini, Simona Truglia, Cristiana Barbati, Marta Vomero, Danilo Sabatinelli, Francesca Morello, Guido Valesini, Fabrizio Conti

**Affiliations:** Dipartimento di Medicina Interna e Specialità Mediche, Reumatologia, Sapienza Università di Roma, Rome, Italy

**Keywords:** Rheumatoid arthritis, Autoimmune rheumatic diseases, Systemic lupus erythematosus, Sjögren syndrome post-translational modifications, Anti-carbamylated proteins antibodies, Anti-citrullinated peptides antibodies, Rheumatoid factor

## Abstract

**Background:**

Antibodies against carbamylated proteins (anti-CarP) have been recently identified in the sera of patients with rheumatoid arthritis (RA). The objective of the study was to evaluate the prevalence, sensitivity and specificity of anti-CarP compared to anti-citrullinated peptide antibodies (ACPA) and rheumatoid factor (RF), replicating the existing data in a large cohort of Italian patients with RA and extending the evaluation to other autoimmune rheumatic diseases (AIRDs).

**Methods:**

Serum samples (n = 607) from 309 patients with RA, 200 disease controls and 98 normal healthy subjects (NHS) were evaluated. Anti-CarP were detected using carbamylated fetal calf serum as the antigen. ACPAs were detected using second-generation ELISA and IgM RF was assessed as part of routine analysis.

**Results:**

Anti-CarP antibodies were detected in 117 patients with RA (34.4%), ACPA in 190 patients (61.4%) and RF in 202 patients (65.3%). Two (2.04%) of the NHS were positive for anti-CarP, one NHS (1.02%) was positive for ACPA and three NHS were positive for RF (3.06%). Among disease controls, anti-CarP antibodies were detected in 33 patients (16.5%), ACPA in 29 patients (14.5%) and RF in 64 patients (32%). In particular, 16.8% of patients with systemic lupus erythematosus and 31.1% of patients with Sjögren syndrome were positive for anti-CarP. The sensitivity of anti-CarP, ACPA and RF was 46.8%, 61.8% and 64.4%, respectively and specificity was 91.95%, 89.93% and 76.51%, respectively.

**Conclusions:**

The present study extends the knowledge of anti-CarP antibodies, confirming previous data on the diagnostic accuracy of anti-CarP in RA in a large cohort of Italian patients. Anti-CarP antibodies demonstrated relatively low sensitivity and slightly higher specificity compared to ACPA and RF. Even if predominantly present in RA, anti-CarP was detected in a variable percentage of patients with other autoimmune rheumatic diseases and their generation could be attributed to the inflammatory status; the clinical relevance of anti-CarP antibodies in these latter patients should be further determined.

## Background

The discovery of autoantibodies in patients with RA facilitated the subgrouping of these patients for more accurate therapeutic management, resulting in more efficient disease control [1]. Beside the well-known rheumatoid factor (RF), anti-citrullinated protein antibodies (ACPA) have been reported to be a very useful diagnostic and prognostic marker of RA [2]. ACPA have remarkable sensitivity for this disease, with high predictive value for RA development and severity [2, 3]. The importance of ACPA in RA was highlighted by the inclusion of ACPA status in the 2010 classification criteria for RA by allowing the division of patients with RA into two major subsets: ACPA-positive and ACPA-negative [4].

Although ACPA have an important role in the diagnosis of RA, there is still a continuous demand for new biomarkers to further improve the early diagnosis of RA and especially its seronegative subgroup [5]. Recently, a new autoantibody system recognising antibodies against carbamylated proteins (anti-CarP) has been described [6] but has not yet been implemented for commercial use.

Initially, Shi et al. identified homocitrulline as the main aminoacid involved in the binding of autoantibodies in seronegative patients with RA [7]. Carbamylation is a chemical reaction mediated by cyanate that modifies lysine residues [5]. Normally the level of cyanate is in equilibrium with urea but specific conditions like inflammation can warp this equilibrium through a myeloperoxidase-dependent mechanism [8, 9]. This leads to the local increase of cyanate levels, thus empowering the degree of carbamylation [10].

Although the high resemblance in structure between citrulline and homocitrulline, inhibition and cohort studies have demonstrated that ACPA and anti-CarP are different and independent antibody subsets that do not cross-react with each other [4, 5]. Unlike ACPA, the presence of anti-CarP has not been associated with HLA-shared epitope (SE) and/or smoking [11]. An interesting finding was the presence of anti-CarP in ACPA-negative patients with RA and their association with increased disease activity [12, 13] and severe joint damage [13, 14]. Moreover, anti-CarP have been detected in patients with arthralgia and their presence has been independently associated with the risk of developing RA [15]. In addition, anti-CarP are present in serum from patients with RA many years before the clinical appearance of the disease [14, 16, 17] and have also been identified in healthy first-degree relatives of patients with RA [18].

Recently, Shi et al. investigated the diagnostic performance of anti-CarP in RA in a large cohort of patients with early arthritis and demonstrated that these antibodies are predominantly detected in RA, but that a small percentage of patients with almost all forms of early arthritis were also anti-CarP-positive [19]. Considering the high prognostic and predictive value that anti-CarP have demonstrated in patients with RA, the aim of this study was to evaluate their prevalence, sensitivity and specificity in a large monocentric cohort of patients with RA compared to other autoimmune rheumatic diseases (AIRDs).

## Methods

### Patient populations

We evaluated a total of 607 frozen stored serum samples from 309 patients with established RA diagnosed according to the 2010 American College of Rheumatology (ACR)/European League Against Rheumatism (EULAR) criteria, and 200 unselected patients with other diseases [systemic lupus erythematosus (SLE), Sjögren syndrome (SS), systemic sclerosis (SSc), and postmenopausal osteoporosis (OP)], who were not matched by gender or age with the RA group, and who were attending outpatient clinics at Sapienza University of Rome. Serum samples from 98 unselected normal healthy subjects (NHS) were also tested.

### Anti-CarP antibody assays

Anti-CarP antibodies were detected by a modified solid phase “home-made” ELISA as described by Shi et al., with some modifications [20] using carbamylated fetal calf serum (FCS) as the antigen. In brief, Nunc Maxisorp plates (Thermo Scientific, Whaltam, MA, USA) were coated overnight at +4 ° C with non-modified FCS and Ca-FCS (10 μg/ml in carbonate bicarbonate buffer). After washing the plates were blocked with phosphate-buffered saline (PBS) 1% bovine serum albumin (BSA) (Sigma-Aldrich, St. Louis, MO, USA) for 6 h at +4 ° C. Subsequently, the wells were incubated with serum from patients, diluted 1/50 in PBS/0.05% tween/BSA 1% overnight at +4 ° C. After four washes the plates were incubated for 2 h at room temperature (RT) with goat polyclonal antihuman IgG alkaline phosphatase conjugated antibodies (Sigma-Aldrich, St. Louis, MO, USA) diluted at 1:1000 in PBS/0.05% tween/BSA 1%. After four washes a solution of paranitrophenyl phosphate tablets in ethanolamine was used for the enzyme reaction and the plates were read at a 405 nm wavelength after 30 minutes at RT. All assays were performed in duplicate and the absorbance of control wells (unmodified FCS) was subtracted to account for non-specific binding. The levels were determined in arbitrary units per millilitre (AU/ml) using a standard curve. The cutoff for anti-CarP antibody ELISA was established as the mean plus three times the standard deviation (SD) of the healthy control.

### Anti-CCP and RF antibody assays

ACPA were detected using a second-generation ELISA (anti-CCP) kit (Delta Biologicals, Italy) while IgM RF was determined as part of routine analysis by immunonephelometry (Behering, Marburg, Germany) according to the manufacturers’ instructions.

### Statistical analysis

The diagnostic performance of ACPA, RF and anti-CarP antibodies assays was determined by receiver operating characteristic (ROC) curve analysis, by plotting sensitivity (%) against 100-specificity (%) at different cutoff values. Diagnostic sensitivity was compared at cutoff levels corresponding to 95% specificity. Dunn’s multiple comparison test was used to compare the levels of anti-CarP antibodies between the different diagnoses and the chi-square test was used to compare the percentage of the patients with other rheumatic conditions who were positive for the these antibodies. *P* values <0.05 were considered statistically significant. Data analysis was performed using SPSS version 20 (SPSS Inc., Chicago, IL, USA).

## Results

We analysed serum from 309 patients with RA (mean age 55.4 ± 13.8 years), who had a mean disease duration of 107 ± 97.6 months; 238 (77%) were female, and 167 (54%) were smokers. Table 1 shows the characteristic of patients with RA and the control groups.

**Table 1:** Characteristic of study participants

Disease (number of patients)	Age (years) (mean + SD)	Female *n*, (%)	Smokers *n*, (%)
RA (309)	55.4 + 13.8	238 (77)	167 (54)
SLE (83)	42.8 + 11.3	57 (68.6)	34 (40.9)
SSC (51)	50.4 + 12.1	51 (100)	10 (19.6)
SS (45)	53.3 + 13.1	30 (66.6)	26 (48.1)
OP (21)	66.1 + 7.2	21 (100)	2 (9.5)
HS (98)	53.1 + 10.4	73 (74.5)	43 (43.9)

*RA* rheumatoid arthritis, *SLE* systemic lupus erythematosus, *SS* Sjögren syndrome, *SSc* systemic sclerosis, *OP* osteoporosis, *HS* healthy subjects

### Anti-CarP, anti-CCP and RF in Patients with RA and controls

The overall prevalence of anti-CarP, RF, and anti-CCP in patients with RA, disease controls and healthy subjects is shown in Table 2.

**Table 2:** Prevalence of anti-CarP, ACPA and RF in patients with RA, disease controls and healthy subjects

Disease (number of patients)	Anti-CarP positive, *n*, (%)	ACPA positive, *n*, (%)	RF positive, n, (%)
RA (309)	117 (34.4)	190 (61.4)	202 (65.3)
NHS (98)	2 (2.04)	1 (1.02)	3 (3.06)
SLE (83)	14 (16.8)	15 (18.07)	19 (22.8)
SS (45)	14 (31.1)	11 (24.4)	23 (51.1)
SSc (51)	3 (5.8)	1 (1.9)	18 (35.2)
OP (21)	2 (9.5)	2 (9.5)	4 (19.04)

*Anti-CarP* anti-carbamylated proteins antibodies, *ACPA* anti-citrullinated peptides antibodies, *RF* rheumatoid factor, *RA* rheumatoid arthritis, *NHS* normal healthy subjects, *SLE* systemic lupus erythematosus, *SS* Sjögren syndrome, *SSc* systemic sclerosis, *OP* osteoporosis

**Fig. 1:**
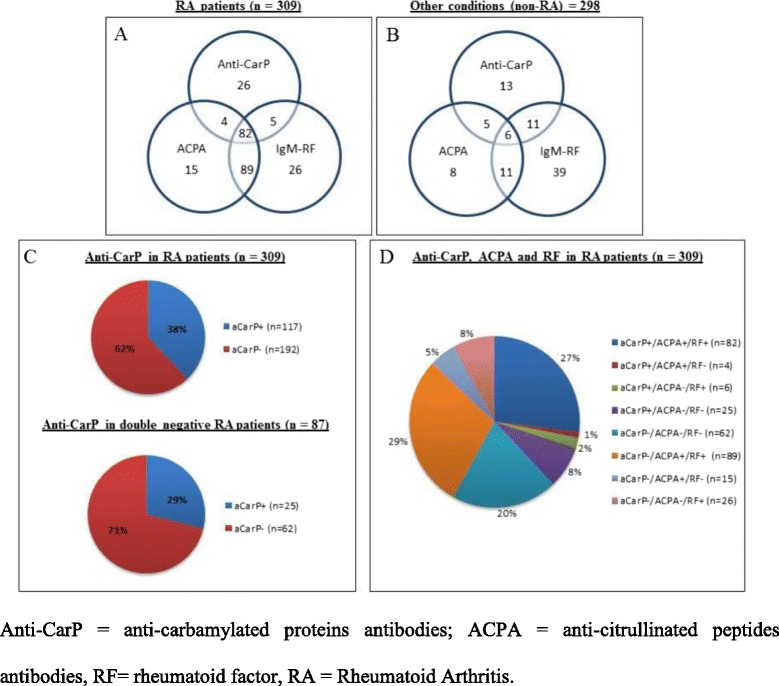
Distribution of antibodies against carbamylated proteins (*anti-CarP*), anti-citrullinated protein antibodies (*ACPA*) and rheumatoid factor (*RF*) in patients with rheumatoid arthritis (*RA*) and disease controls. **a**–**b** Venn diagram showing the relationship between anti-CarP antibodies, ACPA and IgM RF in patients with RA (**a**) and other conditions (non-RA) (**b**). **c** Anti-CarP positivity in the whole RA population and double-negative patients with RA. **d** Distribution of single, double and triple positivity for anti-CarP, ACPA and RF in patients with RA

The prevalence of anti-CarP antibodies was significantly higher in patients with RA compared to healthy controls (*p* = 0.00005) and non-RA disease (*p* < 0.00001 for SLE, SSc and OP) but not compared to SS (*p* = 0.05). Even when considering the disease controls as a whole group, the prevalence of anti-CarP antibodies was still significantly higher in patients with RA (*p* < 0.00001). Figure 1 shows the distribution of anti-CarP, ACPA and IgM-RF in patients with RA (Fig. 1a) and non-RA disease (Fig. 1b); most of the patients with RA were positive for all three antibodies tested (27% of patients) or as combination of two antibodies. Conversely, among the non-RA disease controls only two patients with SLE had double positivity for anti-CarP and ACPA and other two were triple-positive and were diagnosed as having Rhupus; moreover, two patients with SS were triple-positive and one of them was diagnosed as having RA-associated SS. Interestingly, in the subset of seronegative patients with RA, 29% of these patients (25 out of 87) had anti-CarP antibodies (Fig. 1c and d).

When evaluating the association between RA subsets according to antibody positivity and cigarette smoking there was an association with the presence and titre of ACPA (*p* = 0.001) but not with anti-CarP positivity or with RF (Table 3). None of the autoantibodies correlated with disease activity as measured by disease activity score in 28 joints (DAS 28).

**Table 3:** Association between smoking and different RA subsets according to the presence/absence of anti-CarP antibodies, ACPA and RF

Smoking					
Disease subset	Patients, *n*	Never smoked, *n* (%)	Ever smoked, *n* (%)	95% CI	*P* value
Anti-CarP (−)/ACPA (−)/RF (−)	62	31 (50%)	31 (50%)	−2.2–2.2	ns
Anti-CarP (+)/ACPA (+)/RF (+)	82	32 (39%)	50 (61%)	19.7–24.7	0.0001
Anti-CarP (−)/ACPA (+)/RF (+)	89	40 (45%)	49 (55%)	7.7–12.2	0.0001
Anti-CarP (+)/ACPA (−)/RF (−)	26	14 (53.8%)	12 (46.2%)	−4.2–0.2	ns
Anti-CarP (−)/ACPA (+)/RF (−)	15	6 (40%)	9 (60%)	0.7–5.2	0.02
Anti-CarP (−)/ACPA (−)/RF (+)	26	14 (53.8%)	12 (46.2%)	−4.2–0.2	ns
Anti-CarP (+)/ACPA (+)/RF (−)	4	2 (50%)	2 (50%)	−2.2–2.2	ns
Anti-CarP (+)/ACPA (−)/RF (+)	5	3 (60%)	2 (40%)	−3.2–1.2	ns

*RA* rheumatoid arthritis, *Anti-CarP* antibodies against carbamylated proteins, *ACPA* anti-citrullinated protein antibodies, *RF* rheumatoid factor, *ns* not significant

**Fig. 2:**
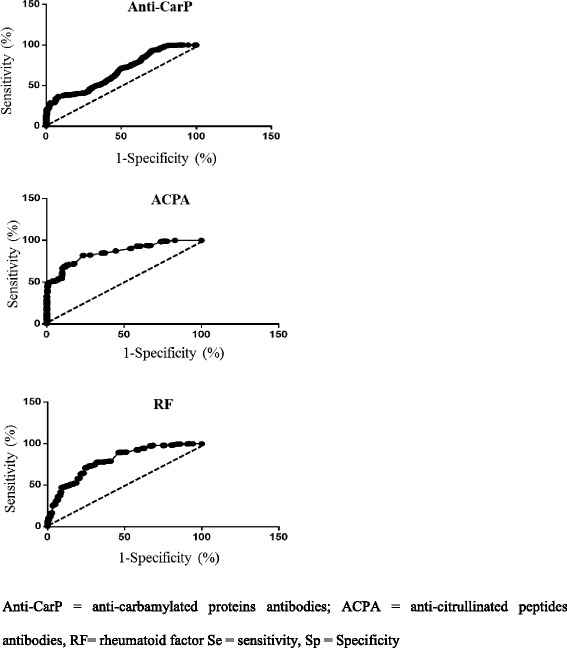
Receiver operating characteristic curve for antibodies against carbamylated proteins (*anti-CarP*), anti-citrullinated protein antibodies (*ACPA*) and rheumatoid factor (*RF*)

The AUC from ROC analysis (Fig. 2) was 0.678 (95% CI 0.636–0.720) for anti-CarP, 0.858 (95% CI 0.830–0.887) for ACPAs and 0.796 (95% CI 0.761–0.831) for RF. Among the three antibodies studied, anti-CarP antibodies had the highest specificity but relatively low sensitivity (91.9 and 46.8, respectively). Table 4 summarizes the diagnostic performance of anti-CarP compared to ACPA and RF.

**Table 4:** Diagnostic performance of anti-CarP antibodies, ACPA and RF

	Sensitivity (95% CI)	Specificity (95% CI)	LR	PPV	NPV
Anti-CarP	46.8 (41.50–52.54)	91.95 (82.25–94.77)	4.581	88%	60%
ACPA	61.8 (56.14–67.25)	89.93 (85.94–93.10)	6.14	85%	62%
RF	64.4 (58.78–69.74)	76.51 (71.28–81.21)	2.742	80%	69%

*Anti-CarP* antibodies against carbamylated proteins, *ACPA* anti-citrullinated protein antibodies, *RF* rheumatoid factor, *LR* likelihood ratio, *PPV* positive predictive value, *NPV* negative predictive value

**Fig. 3:**
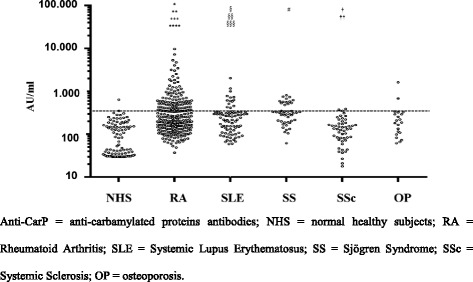
Antibodies against carbamylated proteins (*anti-CarP*) in serum from patients and controls. *Horizontal line* corresponds to cutoff value: *p < 0.0005 for rheumatoid arthritis (*RA*) vs normal healthy subjects (*NHS*) and patients with osteoporosis (*OP*); **p = 0.03 for RA vs systemic lupus erythematosus (*SLE*); ***p = 0.04 for RA vs Sjögren syndrome (*SS*); ****p = 0.004 RA vs systemic sclerosis (*SSc*); ^§^ *p* = 0.00001 for SLE vs NHS; ^§§^ *p* = 0.002 for SLE vs SSc; ^§§§^ *p* = 0.05 for SLE vs OP; ^#^ *p* = 0.00001 for SS vs NHS and SS vs SSc; ^†^ *p* = 0.03 for SSc vs NHS; ^††^ *p* = 0.004 for SSc vs OP

Anti-CarP serum levels in patients with RA and in controls are shown in Fig. 3. When comparing the anti-CarP titre in positive patients we noticed that the levels were significantly higher in patients with RA compared to non-RA conditions (*p* < 0.05 for all conditions) (Fig. 3). Nevertheless, high levels of anti-CarP antibodies were detected even in the few patients with SLE and SS, although these levels were never as high as the levels of anti-CarP antibodies in the patients with RA (Fig. 3). Anti-CarP, ACPA and IgM-RF were not statistically different among the patients with non-RA disease (*p* > 0.05 for all).

## Discussion

In recent years, the importance of early diagnosis and treatment toward more efficient control of the disease, less joint damage and a better outcome has been clearly stated. For this reason, research has mostly focused on identifying biomarkers and specific laboratory tests to be applied in the early detection of RA [1]. The present study analyses prevalence, sensitivity and specificity of anti-CarP antibodies, replicating previously published data in a large, single-centre cohort of Italian patients with RA compared to healthy controls and patients with other AIRDs. For the first time we investigated in a single study the positivity of anti-CarP, even including patients with different systemic AIRDs, which are known to be associated with the production of several autoantibodies.

In this study we confirmed the data on anti-CarP showing the presence of these antibodies in a significant number of patients with RA. Actually, 15 patients were single-positive for ACPA and 26 were single-positive for anti-CarP, confirming the lack of cross-reactivity between anti-citrullinated and anti-homocitrullinated proteins as previously described [6]. Isolated anti-CarP positivity was detected in about one third of patients with RA who were seronegative for ACPA and RF.

The major advantage of simultaneous or alternative detection of a wide range of biomarkers consists in a much detailed classification of RA subtypes, increasing the sensitivity of RA diagnosis [2, 5]. As suggested by the results of previous research [6, 7], in this study we were able to identify different immunological RA subsets based on the presence and/or absence of the three autoantibodies studied (anti-CarP, ACPA and RF) opening a new window of opportunity to more precise disease classification and treatment. When comparing the findings of the present study with previous results, there is a difference in the percentage of triple positivity for anti-CarP, ACPA and RF: indeed, the percentage of triple-positive patients is lower among patients in the Norfolk Arthritis Register and higher in the early arthritis Leiden cohort compared to the present cohort of Italian patients with RA [12, 19]; this discrepancy could result from the different genetic background of the populations studied. Moreover, unlike the results of a longitudinal study, reporting higher disease activity over time in anti-CarP-positive patients [12], the findings of our cross-sectional analysis agree with the lack of association with disease activity reported by others [13].

Unlike citrullination - that is an enzimatically mediated reaction - carbamylation is a chemical reaction that occurs in the presence of a reactive metabolite (cyanate) [6]. Theoretically, every protein can be carbamylated in vivo but the probability of a protein undergoing such modification depends on various parameters such as the number and accessibility of lysine and arginine amino groups and the protein lifespan [7]. As carbamylation is an almost irreversible reaction, it is more likeky to affect long-lived proteins as they may have acquired homocitrulline residues over time [7]. Taken together, these data suggest that even the antibodies produced by these post-translational modification reactions may differ in terms of sensitivity and specificity. Thus, in the present study, we compared the diagnostic performance of anti-CarP antibodies, ACPAs and RF in a large monocentric cohort of patients with RA compared to disease and healthy controls with different AIRDs.

As already mentioned, anti-CarP antibodies were present in a significant percentage in seronegative and seropositive patients with RA. Beside patients with RA, anti-CarP antibodies were present in a small number of patients with SLE and SS; less than one third of these patients also displayed ACPA positivity. These results can be partially attributed to erosive joint disease described in a small percentage of patients with SLE [21]; besides, carbamylation can be enhanced in patients with kidney involvement or in those with active inflammatory status [8]. Similarly, a clear link between antibody production and the degree of inflammation has been shown in patients with SS, as the activity of circulating myeloperoxidase (MPO), which catalyzes the production of cyanate from thiocyanate, seems to be significantly increased [20]. Two studies investigated anti-CarP in patients with SLE and with SS [20, 21]. The detection of a subset of anti-CarP-positive subjects among patients with SLE or SS and joint involvement suggests the possibility of that anti-CarP might serve as a serological marker for erosive arthritis in AIRDs other than RA. However, the clinical and prognostic relevance of these autoantibodies in patients with non-RA needs to be further elucidated.

In diagnostic performance, anti-CarP antibodies had slightly higher specificity but relatively lower sensitivity when compared to ACPA and IgM-RF. On the other hand, the AUC for anti-CarP antibodies in the whole population was 0.678, whereas in the seronegative group it was only 0.565. These data suggest an additional role of anti-CarP positivity in the diagnosis of RA, in agreement with previous studies reporting the presence and prognostic value of anti-CarP antibodies in the RA seronegative subgroup, and correlation with joint destruction [7, 12, 13, 15]. The diagnosis of RA is mostly clinical, and it is supported by serological data. Nonetheless, as we are moving toward the identification of RA in its early phases, biomarkers could help in stratifying subjects at risk of developing overt disease even in pre-clinical phases [4, 5].

## Conclusions

In conclusion, the results of the present study extend the knowledge on anti-CarP providing updated data on prevalence, sensitivity and specificity on a large cohort of Italian patients. Moreover, we confirm that anti-CarP antibodies and ACPA are not always present simultaneously. The positivity of these antibodies in patients with non-RA autoimmune rheumatic disease can be attributed to the inflammatory status and should be addressed in further studies in order to better understand their role in autoimmune diseases.

## Abbreviations

ACPAs: anti-citrullinated protein antibodies
ACR: American College of Rheumatology
AIRDs: autoimmune rheumatic diseases
anti-CarP: antibodies against carbamylated proteins
anti-CCP: anti-cyclic citrullinated peptides
AU: arbitrary units
AUC: area under the curve
BSA: bovine serum albumin
DAS28: disease activity score in 28 joints
ELISA: enzyme-linked immunosorbent assay
EULAR: European League Against Rheumatism
FCS: fetal calf serum
Ig: immunoglobulin
LR: likelihood ratio
NHS: normal healthy subjects
NPV: negative predictive value
OP: osteoporosis
PBS: phosphate-buffered saline
PPV: positive predictive value
RA: rheumatoid arthritis
RF: rheumatoid factor
ROC: receiver operating characteristic
RT: room temperature
SD: standard deviation
SE: shared epitope
SLE: systemic lupus erythematosus
SS: Sjögren syndrome
SSc: systemic sclerosis

## References

[1] Bax M, Huizinga WJ, Toes RE (2014). The pathogenic potential of autoreactive antibodies in rheumatoid arthritis. Semin Immunopathol..

[2] van Venrooij WJ, van Beers JJ, Pruijn GJ (2011). Anti-CCP antibodies: the past the present and the future. Nat Rev Rheumatol..

[3] Valesini G, Gerardi MC, Iannuccelli C, Pacucci VA, Pendolino M, Shoenfeld Y (2015). Citrullination and autoimmunity. Autoimmun Rev.

[4] Aletaha D, Neogi T, Silman AJ, Funovitis J, Felson DT, Binham CO (2010). 2010 rheumatoid arthritis classification criteria: an American College of Rheumatology/European League Against Rheumatism collaborative initiative. Arthritis Rheum..

[5] Trouw LA, Mahler M (2012). Closing the serological gap: promising novel biomarkers for the early diagnosis of rheumatoid arthritis. Autoimmun Rev..

[6] Shi J, Willemze A, Janssen GM, van Veelen PA, Drijfhout JW, Cerami A (2013). Recognition of citrullinated and carbamylated proteins by human antibodies: specificity, cross-reactivity and the “AMC-Shensu” method. Ann Rheum Dis..

[7] Shi J, Knevel R, Suwannalai P, van der Linden MP, Janssen GM, van Veelen PA (2011). Autoantibodies recognising carbamylated proteins are present in sera of patients with rheumatoid arthritis and predict joint damage. Proc Natl Acad Sci USA.

[8] Wang Z, Nicholls SJ, Rodriguez ER, Kummu O, Hörkkö S (2007). Protein carbamylation links inflammation, smoking, uremia and atherogenesis. Nat Med..

[9] Nzeusseu Toukap A, Delporte C, Noyon C, Franck T, Rousseau A, Serteyn D (2014). Myeloperoxidase and its products in synovial fluid of patients with treated or untreated rheumatoid arthritis. Free Radic Res..

[10] Shi J, van Veelen PA, Mahler M, Janssen GM, Drijfhout JW, Huizinga TW (2014). Carbamylation and antibodies against carbmylated proteins in autoimmunity and other pathologies. Autoimmun Rev..

[11] Jiang X, Trouw LA, van Wesemael TJ, Shi J, Bengtsson C, Källberg H (2014). Anti-CarP antibodies in two large cohorts of patients with rheumatoid arthritis and their relationship to genetic risk factors, cigarette smoking and other autoantibodies. Ann Rheum Dis..

[12] Humphreys JH, Verheul MK, Barton A, MacGregor AJ, Lunt M, Toes RE (2016). Anti-carbamylated protein antibodies are associated with long-term disability and increased disease activity in patients with early inflammatory arthritis: results from the Norfolk Arthritis Register. Ann Rheum Dis..

[13] Yee A, Webb T, Seaman A, Infantino M, Meacci F, Manfredi M (2015). Anti-CarP antibodies as promising markers to measure joint damage and disease activity in patients with rheumatoid arthritis. Immunol Res..

[14] Brink M, Verheul MK, Rönnelid J, Berglin E, Holmdahl R, Toes RE (2015). Anti-carbamylated protein antibodies in the pre-symptomatic phase of rheumatoid arthritis, their relationship with multiple anti-citrulline peptide antibodies and association with radiological damage. Arthritis Res Ther..

[15] Shi J, van de Stadt LA, Levarht EW, Huizinga TW, Toes RE, Trouw LA (2013). Anti-carbamylated protein antibodies are present in arthralgia patients and predict the development of rheumatoid arthritis. Arthritis Rheum..

[16] Shi J, van de Stadt LA, Levarht EW, Huizinga TW, Hamann D, van Schaardenburg D (2014). Anti-carbamylated protein antibodies precede the onset of rheumatoid arthritis. Ann Rheum Dis..

[17] Gan RW, Trouw LA, Shi J, Toes RE, Huizinga TW, Demoruelle MK (2015). Anti-carbamylated protein antibodies are present prior to rheumatoid arthritis and are associated with its future diagnosis. J Rheumatol..

[18] Alessandri C, Bartosiewicz I, Pendolino M, Mancini R, Colasanti T, Pecani A (2015). Anti-Carbamylated protein antibodies in unaffected first-degree relatives of rheumatoid patients: lack of correlation with anti-cyclic citrullinated protein antibodies and rheumatoid factor. Clin Exp Rheumatol..

[19] Shi J, van Steenbergen HW, van Nies JA, Levarht EW, Huizinga TW, van der Helm-van Mil AH (2015). The specificity of anti-carbamylated protein antibodies for rheumatoid arthritis in a setting of early arthritis. Arthritis Res Ther..

[20] Kastbom A, Wallin P, Ziegelasch M, Skogh T, Trouw L, Sjöwall C. Anti-Carbamylated Protein Antibodies Identify Systemic Lupus Erythematosus Patients with Erosive Arthritis: Analysis of a Regional Swedish Register [abstract]. Arthritis Rheumatol. 2015; 67 (suppl 10). http://acrabstracts.org/abstract/anti-carbamylated-protein-antibodies-identify-systemic-lupus-erythematosus-patients-with-erosive-arthritis-analysis-of-a-regional-swedish-register/. Accessed 20 Nov 2016.

[21] Berqum B, Koro C, Delaleu N, Solheim M, Hellward A, Binder V (2016). Antibodies against carbamylated proteins are present in primary Sjogren’s syndrome and are associated with disease severity. Ann Rheum Dis.

