# Erratum to: Prevalence, sensitivity and specificity of antibodies against carbamylated proteins in a monocentric cohort of patients with rheumatoid arthritis and other autoimmune rheumatic diseases

**DOI:** 10.1186/s13075-016-1205-9

**Published:** 2016-12-16

**Authors:** Arbi Pecani, Cristiano Alessandri, Francesca Romana Spinelli, Roberta Priori, Valeria Riccieri, Manuela Di Franco, Fulvia Ceccarelli, Tania Colasanti, Monica Pendolino, Riccardo Mancini, Simona Truglia, Cristiana Barbati, Marta Vomero, Danilo Sabatinelli, Francesca Morello, Guido Valesini, Fabrizio Conti

**Affiliations:** Dipartimento di Medicina Interna e Specialità Mediche, Reumatologia, Sapienza Università di Roma, Rome, Italy

## Abstract

Anti-CarP antibodies were detected in 117 patients with RA (37.9%), ACPA in 190 patients (61.4%) and RF in 202 patients (65.3%). Two (2.04%) of the NHS were positive for anti-CarP, one NHS (1.02%) was positive for ACPA and three NHS were positive for RF (3.06%). Among disease controls, anti-CarP antibodies were detected in 33 patients (16.5%), ACPA in 29 patients (14.5%) and RF in 64 patients (32%). In particular, 16.8% of patients with systemic lupus erythematosus and 31.1% of patients with Sjögren syndrome were positive for anti-CarP. The sensitivity of anti-CarP, ACPA and RF was 46.8, 61.8 and 64.4%, respectively and specificity was 91.95, 89.93 and 76.51%, respectively.

## Erratum

Unfortunately, after publication of this article [1], it was noticed that the percentage of patients listed as RA positive was incorrect in two locations. In the ‘Results’ section of the abstract and in row 1, column 2 of Table 2, the percentage was listed as 34.4%. The actual percentage should be 37.9%. The corrected ‘Results’ section of the abstract can be seen above and the corrected Table 2 can be seen below.

**Table 2:** Prevalence of anti-CarP, ACPA and RF in RA patients, disease controls and healthy subjects

Disease (No of patients)	Anti-CarP positive n, (%)	ACPA positive n, (%)	RF positive n, (%)
RA (309)	117 (37.9)	190 (61.4)	202 (65.3)
NHS (98)	2 (2.04)	1 (1.02)	3 (3.06)
SLE (83)	14 (16.8)	15 (18.07)	19 (22.8)
SS (45)	14 (31.1)	11 (24.4)	23 (51.1)
SSc (51)	3 (5.8)	1 (1.9)	18 (35.2)
OP (21)	2 (9.5)	2 (9.5)	4 (19.04)

*Anti-CarP* anti-carbamylated proteins antibodies, *ACPA* anti-citrullinated peptides antibodies, *RF* rheumatoid factor, *RA* Rheumatoid Arthritis, *NHS* Normal Healthy Subjects, *SLE* Systemic Lupus Erythematosus, *SS* Sjögren Syndrome, *SSc* Systemic Sclerosis, *OP* osteoporosis
